# The value of a metabolic and immune-related gene signature and adjuvant therapeutic response in pancreatic cancer

**DOI:** 10.3389/fgene.2024.1475378

**Published:** 2025-01-03

**Authors:** Danlei Ni, Jiayi Wu, Jingjing Pan, Yajing Liang, Zihui Xu, Zhiying Yan, Kequn Xu, Feifei Wei

**Affiliations:** Department of Oncology, The Third Affiliated Hospital of Nanjing Medical University, Changzhou, China

**Keywords:** metabolism and immune-related gene, value, PDAC, prognosis, adjuvant therapy

## Abstract

**Background:**

Pancreatic ductal adenocarcinoma (PDAC) is a highly aggressive malignancy characterized by a dismal prognosis. Treatment outcomes exhibit substantial variability across patients, underscoring the urgent need for robust predictive models to effectively estimate survival probabilities and therapeutic responses in PDAC.

**Methods:**

Metabolic and immune-related genes exhibiting differential expression were identified using the TCGA-PDAC and GTEx datasets. A genetic prognostic model was developed via univariable Cox regression analysis on a training cohort. Predictive accuracy was assessed using Kaplan-Meier (K-M) curves, calibration plots, and ROC curves. Additional analyses, including GSAE and immune cell infiltration studies, were conducted to explore relevant biological mechanisms and predict therapeutic efficacy.

**Results:**

An 8-gene prognostic model (AK2, CXCL11, TYK2, ANGPT4, IL20RA, MET, ENPP6, and CA12) was established. Three genes (AK2, ENPP6, and CA12) were associated with metabolism, while the others were immune-related. Most genes correlated with poor prognosis. Validation in TCGA-PDAC and GSE57495 datasets demonstrated robust performance, with AUC values for 1-, 3-, and 5-year OS exceeding 0.7. The model also effectively predicted responses to adjuvant therapy.

**Conclusion:**

This 8-gene signature enhances prognostic accuracy and therapeutic decision-making in PDAC, offering valuable insights for clinical applications and personalized treatment strategies.

## Introduction

Pancreatic ductal adenocarcinoma (PDAC) is a leading cause of cancer mortality, with a 5-year overall survival rate of merely 11% (Siegel et al., 2024). Late diagnosis due to the lack of early symptoms means that most cases (about 80%) are unresectable at presentation (Ma et al., 2019; McGuigan et al., 2018). Even with surgery, more than 80% of patients experience recurrence or metastasis (Barnes et al., 2019; Huang et al., 2019). As a result, comprehensive treatment following multidisciplinary management should be prioritized alongside surgical intervention. Additionally, a small percentage of patients unexpectedly survive beyond 5 years, but the factors contributing to this prolonged survival remain unknown.

Reprogramming cellular energy metabolism, a hallmark of cancer, has garnered increasing attention over the past decade. In pancreatic cancer, malignant cells extensively alter the metabolic processing of key nutrients, including glucose, amino acids, and lipids, to support their survival, proliferation, and growth (Halbrook and Lyssiotis, 2017; Qin et al., 2020). A growing number of studies have demonstrated that the metabolic characteristics of pancreatic cancer present promising therapeutic opportunities for novel and personalized treatments (Martinez-Outschoorn et al., 2017; Mehla and Singh, 2020). For instance, Zheng et al. discovered that the Fzd5-mediated Wnt/β-catenin signaling pathway in RNF43-mutant PDAC regulates tumor growth in both *in vitro* and *in vivo* settings by modulating cholesterol levels. Furthermore, they found that 25-hydroxysterols could have therapeutic potential for PDAC by blocking the fzd5-cholesterol interaction (Zheng et al., 2022). Another study revealed that pancreatic cancer cells with low expression of carbonic anhydrase 12 (CA12) can regulate the oxidative stress response through the NF-κB signaling pathway, making the cancer cells more susceptible to Auranofin treatment (Deben et al., 2024). Additionally, an increasing number of studies have demonstrated that T-cell-mediated immunotherapy can be optimized by modulating cellular metabolism (Kishton et al., 2017). Immune checkpoint inhibitors have also shown the ability to enhance lymphocyte metabolism in tumors and augment their antitumor effects. Therefore, considering the connection between metabolism and immunotherapy, integrating metabolic modulation into conventional immunotherapy is a viable strategy (Chang et al., 2015; Sengupta et al., 2010).

Existing prognostic models, while helpful, fail to adequately link genetic signatures to treatment efficacy. This study aims to develop a robust model that predicts both prognosis and therapeutic outcomes in PDAC.

## Materials and methods

### PDAC dataset collection

The TCGA database was utilized to retrieve the latest RNA sequencing data and clinical follow-up information for 183 PDAC patients (Supplementary Table S1). Corresponding mRNA expression profiles and clinical data for 88 normal samples were acquired from the Genotype-Tissue Expression (GTEx) database.

### Human tissues

Tissue samples were collected from patients who underwent surgery from January 2023 to March 2024 at The Third Affiliated Hospital of Nanjing Medical University. The study protocol was approved by the Institutional Ethics Review Board of the hospital. Fifteen pairs of PDAC and their adjacent non-tumor tissues, totaling 30 cases, were selected for testing.

### Establishment of the 8-gene signature

Only patients with a follow-up period of longer than 1 month were included for the survival analysis. Prognostic genes were identified using univariable Cox regression analysis (*p* < 0.001). Patients were then randomized into training and test sets. Lasso Cox regression analysis was used to further select prognostic genes for OS in PDAC patients. The prognostic gene signature was then constructed as a linear combination of the regression coefficients of the lasso Cox regression model coefficients (β) and their mRNA expression (Tibshirani, 1997; Wang et al., 2022). Risk score = (β mRNA1 * mRNA1 expression level) + (β mRNA2 * mRNA2 expression level) + (βmRNA3 * mRNA3 expression level) +⋯+ (β mRNAn * mRNAn expression level). Patients were classified into high- and low-risk groups based on optimal risk score thresholds calculated using the “Survminer” R package. The predictive value of prognostic genetic signature for OS was assessed by the time-dependent ROC curve. Survival differences between high- and low-risk groups were compared using K-M survival curves combined with log-rank tests using the R package (Heagerty et al., 2000). The predictive value of prognostic genetic signature was then further investigated in the test cohort and the full cohort.

### External validation of the 8-gene signature

The GSE14520 dataset was downloaded from the GEO database (https://www.ncbi.nlm.nih.gov/geo/). Risk scores were calculated for each included patient using the same prognostic gene model. Next, ROC curves and K-M curves were used to test the predictive value of prognostic gene signature.

### Independent prognostic role of the gene signature

To investigate whether prognostic genes could be independent of other clinical parameters (including gender, age, tumor grade, and TNM stage), Cox regression modeling method was used for univariate and multivariate analyses. *p* < 0.05 was considered as statistically significant difference.

### Building and validating a predictive nomogram

Incorporating all independent prognostic factors identified by multivariate Cox regression analysis, a nomogram was constructed to investigate the probability of 0.5-, 1-, and 2-year OS occurring in PDAC (Iasonos et al., 2008). The calibration curve of the nomogram was plotted to compare the nomogram prediction probabilities against the observed rates. Decision curve analysis (DCA) (Vickers and Elkin, 2006) was used to compare the nomogram containing all factors with the nomogram containing only one independent prognostic factor. Calculate the best model.

### Gene set enrichment analysis

To explore the underlying molecular mechanisms of the constructed prognostic gene signature, GSEA (Gene Set Enrichment Analysis) (Subramanian et al., 2005) was used to look for KEGG enrichment terms, and FFDR < 0.05 was considered significantly enriched.

### Estimation of tumor immune infiltration

The relative proportions of different immune cell infiltrates were estimated using the deconvolution algorithm CIBERSORT (Binbin et al., 2018). The number of permutations was set to 1000, and *p* < 0.05 was considered significant.

### Immunohistochemical staining

Tissue sections were incubated with affinity-purified anti-AK2 antibody (Absin Bioscience Inc., Shanghai, China, abs111556) for 2 h. The antigen-antibody complexes were visualized using diaminobenzidine (DAB) as the chromogen. Following DAB staining, the sections were counterstained with hematoxylin to visualize cell nuclei. The slides were then dehydrated through a graded ethanol series, cleared in xylene, and mounted with coverslips. Quantitative analysis of AK2 staining was performed using ImageJ software.

### Statistical analysis

All the statistics, except descriptive analysis, were conducted by R language (version 4.2.1) Data are presented as mean ± SD. Comparisons between groups were made using Student’s t-test or the Mann-Whitney *U* Test. When the *p* < 0.05, results were considered statistically significant.

## Results

### Identification of differentially expressed metabolic and immune related gene and molecular subtypes

**FIGURE 1:**
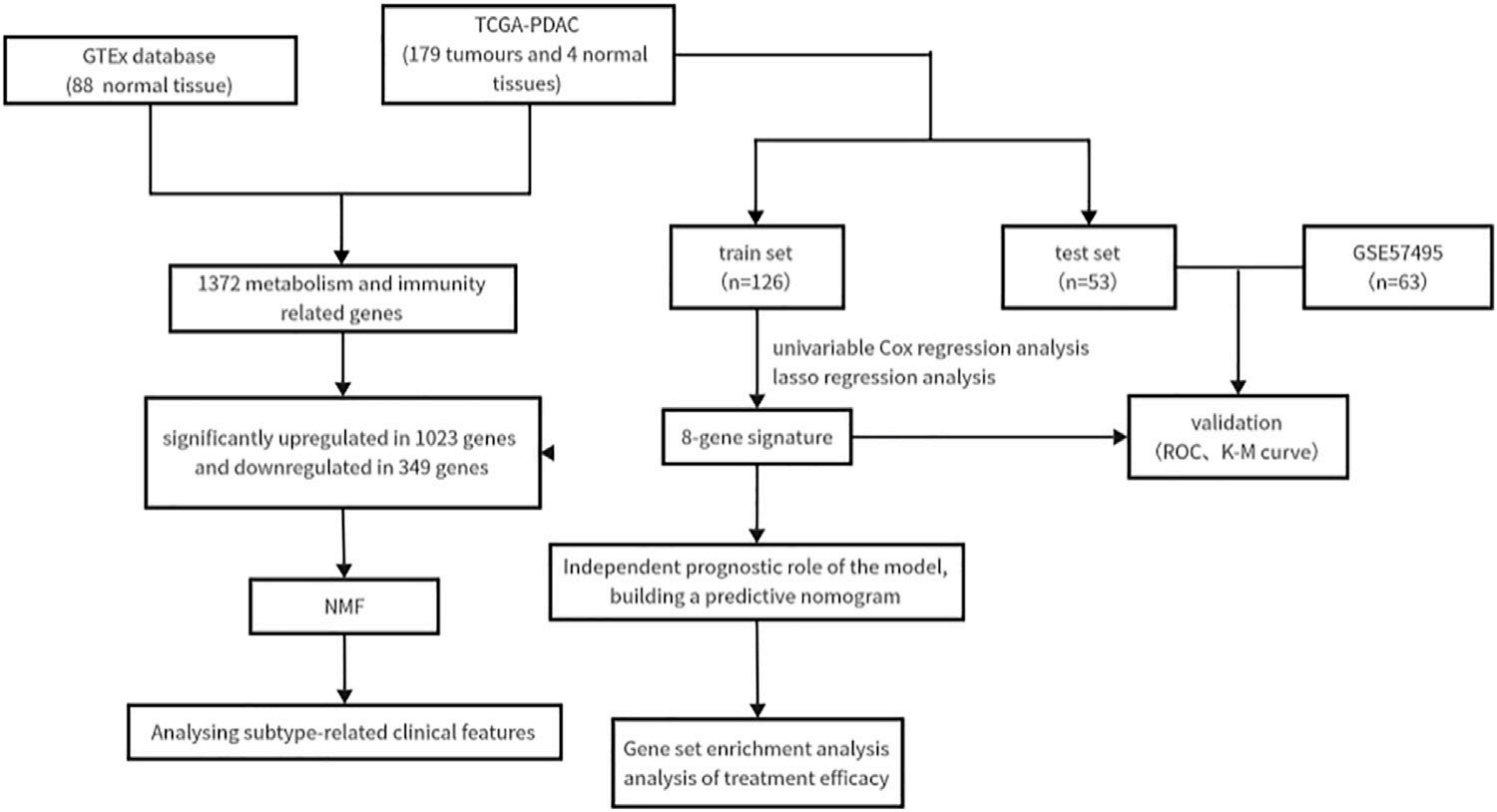
The flow chart showing the scheme of this study.

**FIGURE 2:**
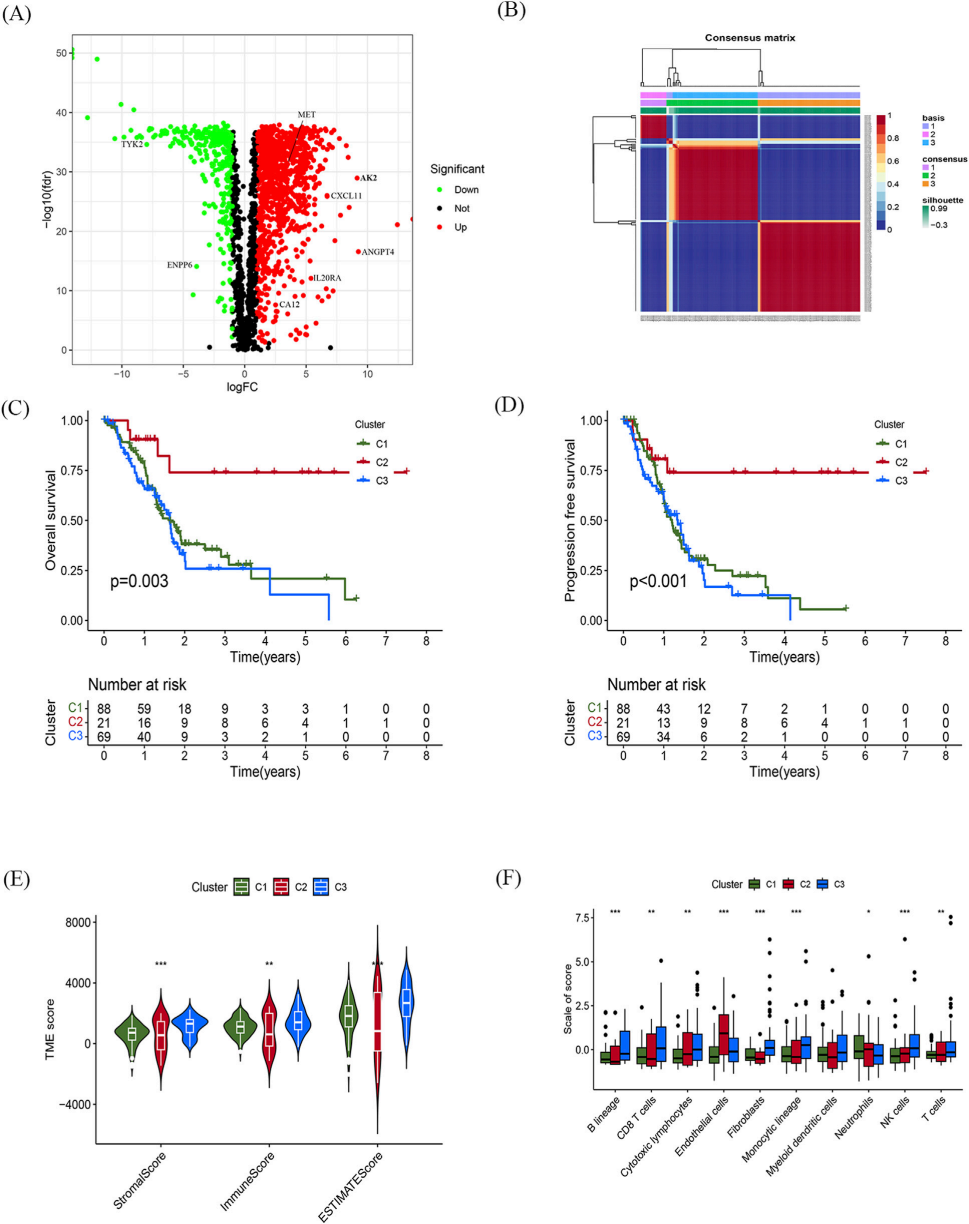
Identification of differentially expressed metabolic and immune related gene and molecular subtypes **(A)** Volcano plot showing significant differential genes, |logFC| > 1.0, fdr < 0.05. **(B)** NMF cluster analysis of typing heatmaps. **(C,D)** K-M survival curves of OS **(C)** and PFS **(D)** for different typing samples. **(E,F)** Violin plots of tumour microenvironment scores **(E)** and differences in immune cell infiltration **(F)** for different typing, green represents C1, red represents C2, blue represents C3, *p < 0.05, **p < 0.01, ***p < 0.01.

The study followed the flow chart outlined in Figure 1. A total of 1372 metabolic and immune-related genes displayed significant differences in mRNA expression levels between tumor tissues (n = 179) and normal tissues (n = 92) (Supplementary Table S1). The corresponding heatmap can be seen in Supplementary Figure S1. Among these genes, 1023 were found to be significantly upregulated and 349 were found to be significantly downregulated in mRNA levels (Figure 2A).

Based on indicators such as cophenetic, dispersion, and silhouette, it was determined that the optimal number of clusters for classification was three (Figure 2B). Supplementary Figure S1 illustrates the expression of relevant genes within these three subclasses.

Furthermore, the prognostic relationship between the three groups was analyzed. The results indicated that the C2 subtype exhibited the most favorable prognosis. Meanwhile, there was no statistically significant difference in the prognosis between the C1 and C2 subtypes. However, there was a significant difference observed among the three groups in terms of both progression-free survival (PFS) and OS (*p* < 0.05; Figures 2C, D).

To assess the tumor microenvironment of the three subtypes, scoring was performed using ESTIMATE (Figure 2E). The results revealed significant discrepancies in immune and stromal scores between the three subtypes, with the C2 subtype displaying the lowest scores in both categories. Additionally, the infiltration of immune cells within the three subtypes was analyzed using the MCP counter algorithm (Figure 2F). The findings showed that the C2 subtype was primarily infiltrated by centrocytes and endothelial cells, while B lymphocytes, CD8^+^ T cells, and fibroblasts predominantly infiltrated the C1 and C3 subtypes.

### Construction and validation of the prognostic gene signature

**FIGURE 3:**
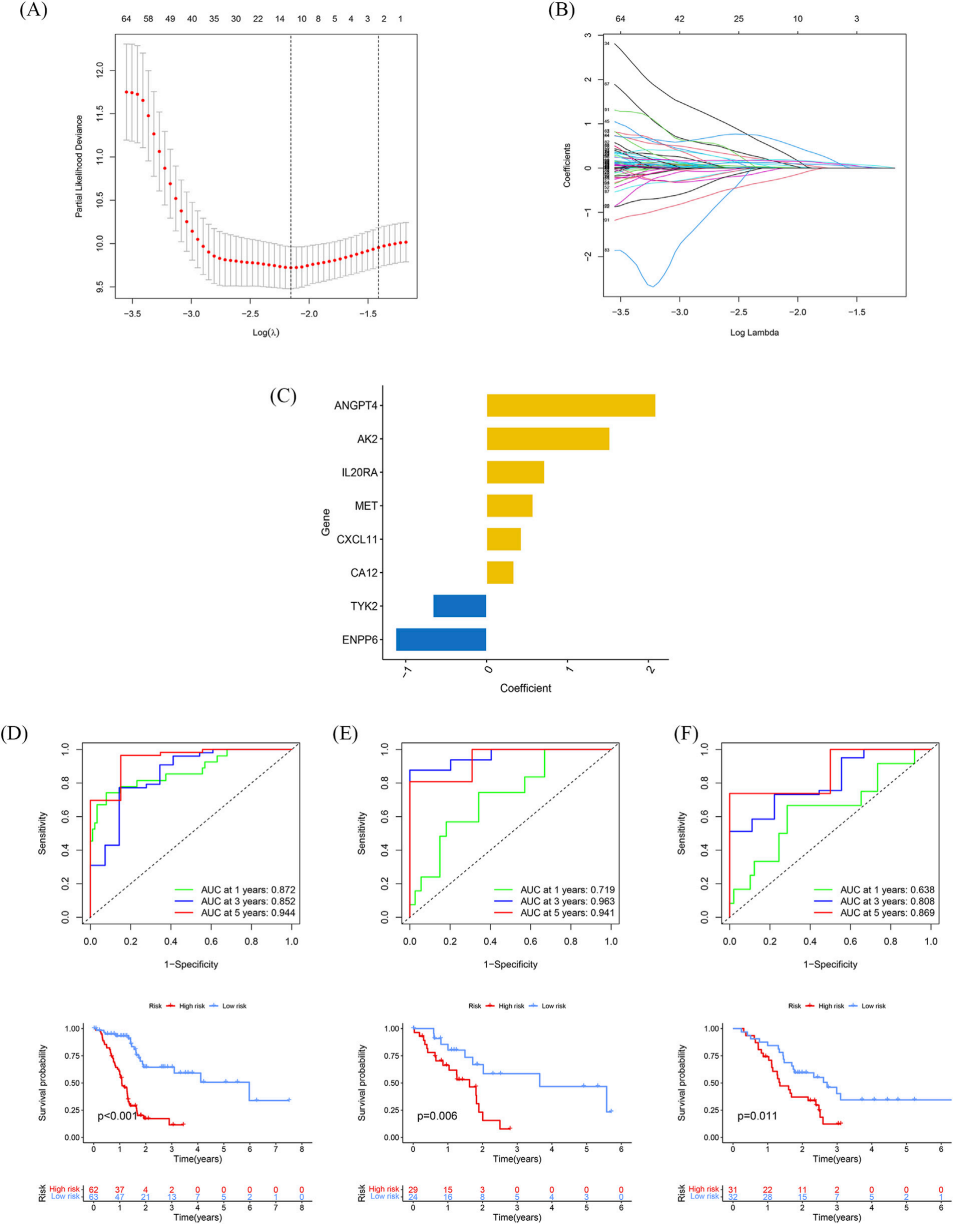
Construction and validation of the prognostic gene signature **(A)** Trajectories of the respective variables, with the horizontal coordinates indicating the logarithmic values of the independent variable lambda and the vertical coordinates indicating the coefficients of the independent variables. **(B)** Confidence intervals at different values of lambda. **(C)** Histogram of the 6-gene prognostic model with vertical coordinates indicating prognostic genes and horizontal coordinates indicating gene coefficients. **(D–F)** Time-dependent ROC analyses and Kaplan-Meier analyses for TCGA-train **(D)**, TCGA-test **(E)**, and GSE57495 **(F)**.

Patients with a follow-up period longer than 1 month were included in the follow-up survival analysis. These patients were then randomly assigned in a 7:3 ratio to a training set (n = 126) and a test set (n = 53). The baseline characteristics of the patients are presented in Table 1. No significant differences in clinical parameters were found between the training and test sets. Univariate Cox regression models were used to identify genes that were significantly associated with OS. Subsequently, lasso regression analysis was performed on the training set to further narrow down the genes of interest (Figures 3A, B). Eight genes were identified: AK2, CXCL11, TYK2, ANGPT4, IL20RA, MET, ENPP6, and CA12 (Figure 3C). These genes were then used to construct a prognostic gene signature.

**TABLE 1:** Baseline characteristics of TCGA-PDAC patients.

Covariates	Type	Total	Test	Train	*p*-value
Age	≤65	94 (52.81%)	30 (56.6%)	64 (51.2%)	0.6197
Age	>65	84 (47.19%)	23 (43.4%)	61 (48.8%)	
Gender	FEMALE	80 (44.94%)	29 (54.72%)	51 (40.8%)	0.1231
Gender	MALE	98 (55.06%)	24 (45.28%)	74 (59.2%)	
Grade	G1	31 (17.42%)	13 (24.53%)	18 (14.4%)	0.3503
Grade	G2	95 (53.37%)	26 (49.06%)	69 (55.2%)	
Grade	G3	48 (26.97%)	14 (26.42%)	34 (27.2%)	
Grade	G4	2 (1.12%)	0 (0%)	2 (1.6%)	
Grade	unknow	2 (1.12%)	0 (0%)	2 (1.6%)	
Stage	Stage I	21 (11.8%)	6 (11.32%)	15 (12%)	0.9959
Stage	Stage II	147 (82.58%)	43 (81.13%)	104 (83.2%)	
Stage	Stage III	3 (1.69%)	1 (1.89%)	2 (1.6%)	
Stage	Stage IV	4 (2.25%)	1 (1.89%)	3 (2.4%)	
Stage	unknow	3 (1.69%)	2 (3.77%)	1 (0.8%)	
T	T1	7 (3.93%)	2 (3.77%)	5 (4%)	0.9713
T	T2	24 (13.48%)	6 (11.32%)	18 (14.4%)	
T	T3	142 (79.78%)	42 (79.25%)	100 (80%)	
T	T4	3 (1.69%)	1 (1.89%)	2 (1.6%)	
T	unknow	2 (1.12%)	2 (3.77%)	0 (0%)	
M	M0	80 (44.94%)	28 (52.83%)	52 (41.6%)	1
M	M1	4 (2.25%)	1 (1.89%)	3 (2.4%)	
M	unknow	94 (52.81%)	24 (45.28%)	70 (56%)	
N	N0	49 (27.53%)	13 (24.53%)	36 (28.8%)	0.7265
N	N1	124 (69.66%)	38 (71.7%)	86 (68.8%)	
N	unknow	5 (2.81%)	2 (3.77%)	3 (2.4%)	

The risk score for each patient, based on the expression levels of these eight genes, was calculated. Patients were stratified into high-risk and low-risk groups based on the optimal risk score threshold identified through the “Survminer” R package. Notably, the high-risk group exhibited markedly worse OS compared to the low-risk group, underscoring its association with poor prognosis (Figure 3D). The prognostic ability of the 8-gene signature was evaluated using ROC curves and K-M curves for 1-, 3-, and 5-year survival in both the test set and the training set. The area under the ROC curve (AUC) for the training set was 0.715, 0.963, and 0.941, respectively, while for the test set, the AUCs were 0.872, 0.852, and 0.944 (Figures 3D, E). To validate the predictive value of the prognostic model, the risk scores were calculated for patients in the GSE57495 dataset (Supplementary Table S2) using the same formula. Consistent with the results from the TCGA cohort, patients in the high-risk group had significantly poorer OS compared to those in the low-risk group (*p* = 0.011). The AUCs for 1-, 3-, and 5-year OS in the GSE57495 dataset were 0.638, 0.808, and 0.869, respectively (Figure 3F). In summary, this 8-gene signature accurately predicts overall survival in patients with PDAC.

### Independent prognostic role of the model and building a predictive nomogram

**FIGURE 4:**
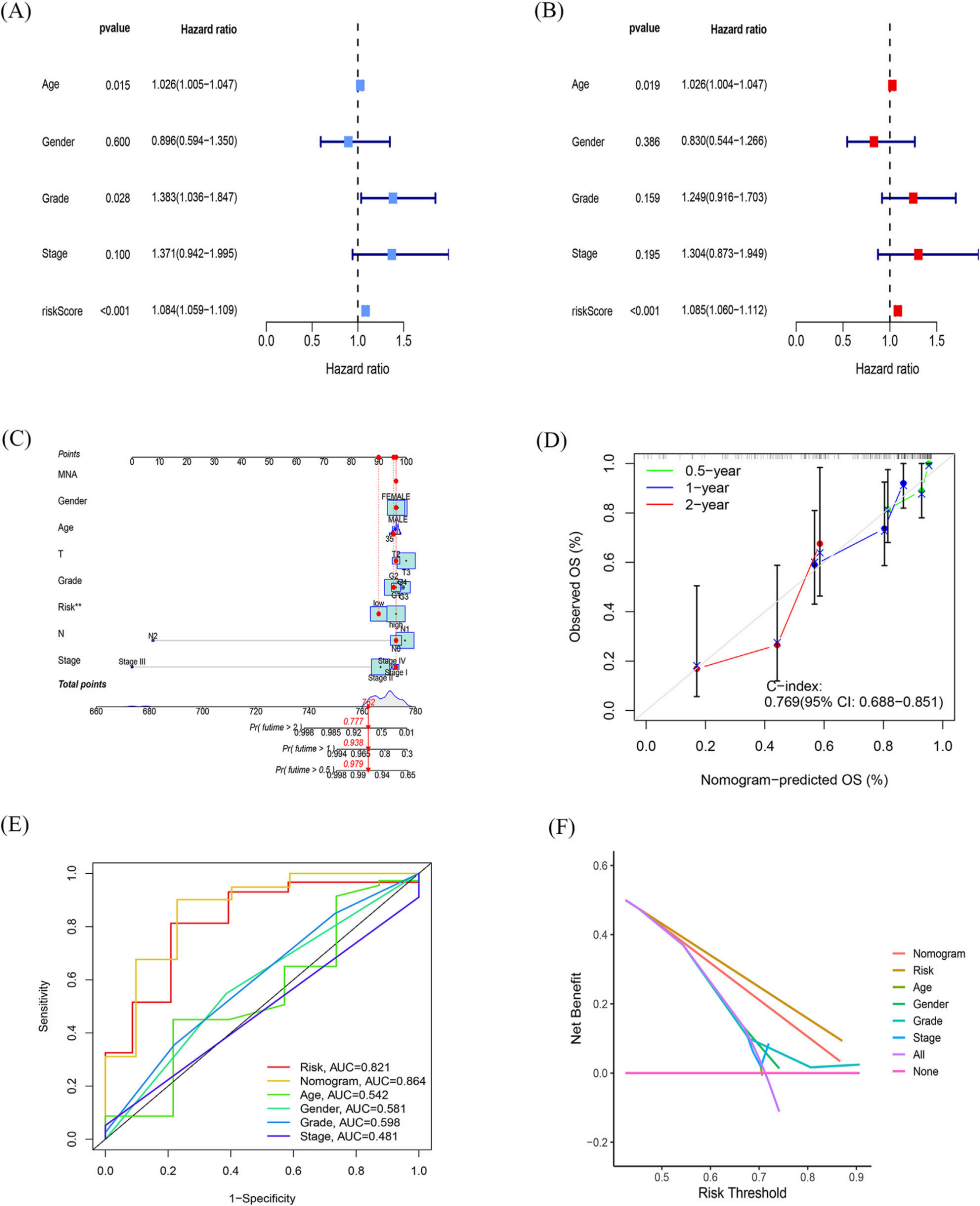
Independent prognostic role of the model and building a predictive nomogram **(A, B)** Forest plots of univariate **(A)** and multivariate **(B)** Cox regression analyses. **(C)** Nomogram based on age, sex, age, TNM stage, and risk score. **(D)** Correction curves for nomograms predicting 6-month, 1-year, and 2-survival rates. **(E)** AUC values for different clinical features, risk scores, and nomograms. **(F)** Decision curve analysis (DCA) for different clinical characteristics, sub-risk scores, and Nomogram, with the *x*-axis representing the threshold probability of mortality and the *y*-axis representing the net benefit.

To determine the independence of this model in clinical application, we utilized clinical information from the TCGA cohort. We calculated the associated hazard ratios (HRs), 95% confidence intervals (CIs) of HRs, and *p*-values through univariate and multivariate Cox regression analyses. We systematically analyzed clinical factors including patient age, gender, grade, clinical stage, and risk score. The results of the univariate Cox regression analysis indicated that clinical factors such as risk score, age, and grade were independent prognostic factors for OS (Figure 4A). However, the multivariate Cox regression analysis showed that only risk score (HR = 1.085, 95% CI 1.060–1.112, *p* < 0.05) and age were independent prognostic risk factors (Figure 4B). These findings suggest that this model demonstrates good predictive performance in clinical applications.

Next, we constructed a nomogram (Figure 4C) to predict 6-month, 1-year, and 2-year OS in PDAC patients. The nomogram incorporated the patient age, gender, grade, clinical stage, and risk score. The calibration plot indicated that the nomogram (combined model) may slightly underestimate or overestimate mortality. The C-index for the combined model was 0.769 (95% CI 0.688–0.851; Figure 4D) and the AUC for the nomogram was 0.864. Comparatively, the combined model exhibited the largest AUC when compared to nomograms that only included age, sex, grading, TNM, or prognostic gene signature (Figure 4E). The DCA showed that the combined model for OS offered the best net benefit (Figure 4F). In conclusion, these results suggest that the nomogram constructed by the combined model may be the most effective tool for predicting short-term survival in PDAC patients. This stands in contrast to nomograms constructed using a single prognostic factor and may provide valuable insights for clinical management.

### Differentiating performance of the prognostic signature

**FIGURE 5:**
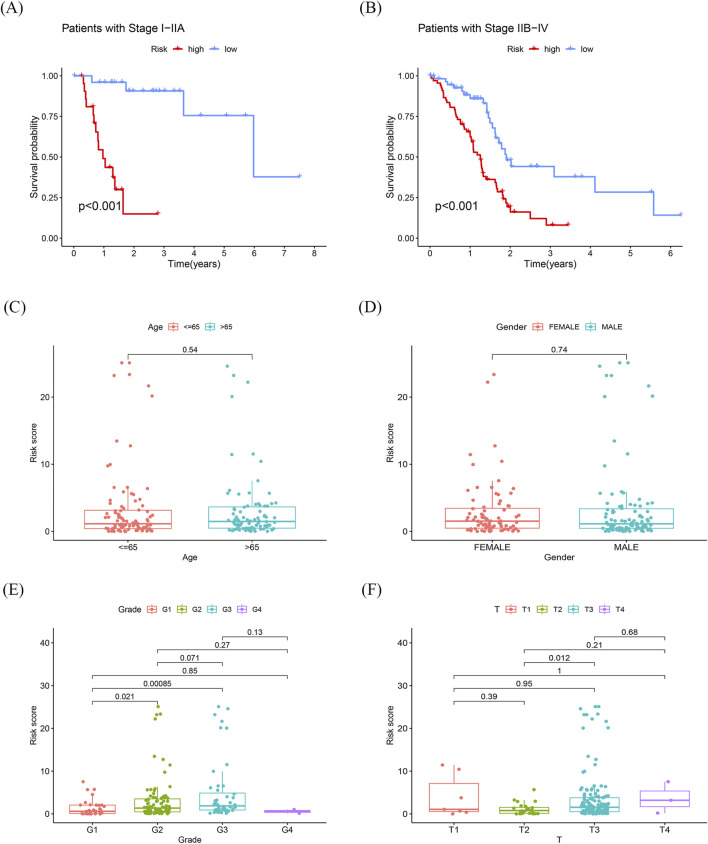
Differentiating performance of the prognostic signature **(A)** K-M curves of sub-risk score for patients with stage I-IIA pancreatic cancer. **(B)** K-M curves of sub-risk score for patients with stage IIB-IV pancreatic cancer. **(C, D)** Differences in prognostic models with age and race were not statistically significant. **(E, F)** Statistically significant differences in prognostic models associated with grading and T-staging.

The risk scores of PDAC patients with different clinical features were compared to explore the diagnostic capability of the prognostic signature. Both early and advanced stage patients with high-risk scores had significantly poorer OS (*p* < 0.001; Figures 5A, B). Unlike age and gender, patients with a higher tumor grade had significantly higher risk scores (Figures 5C–E). Moreover, subgroup analyses of different stages also demonstrated modest diagnostic power (Figure 5F). These results collectively indicate the great potential of this 8-gene signature in the differential diagnosis of PDAC.

### Gene set enrichment analysis and validation of the AK2

**FIGURE 6:**
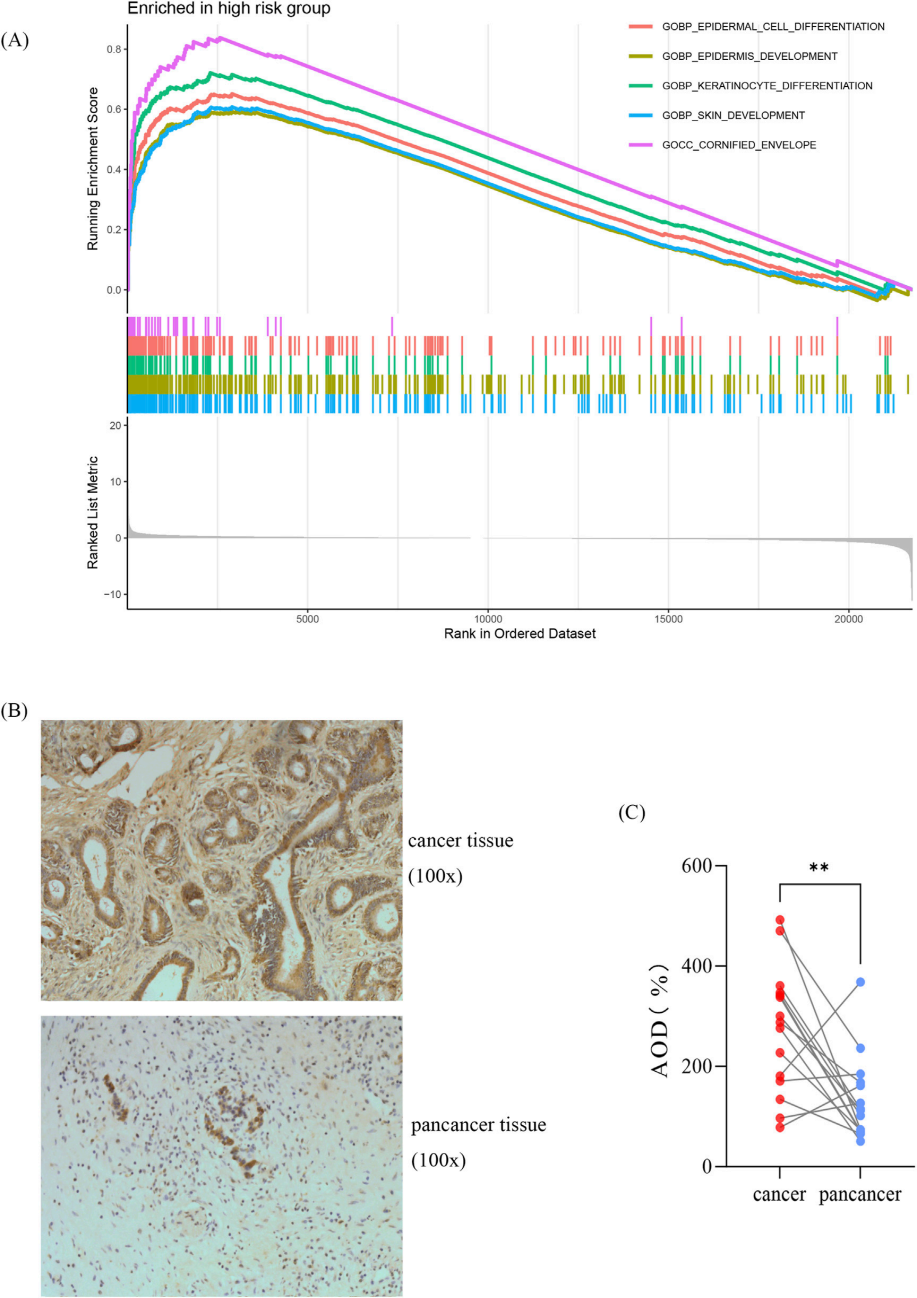
Gene set enrichment analysis and validation of the AK2 **(A)** GO-based GSEA analysis for the high-risk group. **(B)** Representative immunohistochemical images. **(C)** Statistical analysis showing relative levels of AK2 in 15 paired tumor and adjacent samples. Statistical analysis was performed by paired Student’s t-test, *p < 0.05, **p < 0.01, ***p < 0.01.

To investigate the potential molecular mechanisms of the signature, we performed GSEA by comparing the high-risk group with the low-risk group in the TCGA cohort. In the high-risk group, the enriched GO terms primarily focused on cell proliferation and differentiation (Figure 6A). Among the eight genes in this model, AK2 plays a vital role in AK-AMP-AMPK signaling, cell proliferation, and energy transfer in cellular processes. Furthermore, studies have shown that AK2 overexpression may influence the sensitivity of tumor cells to adjuvant therapy (Cai et al., 2021). Consequently, we chose AK2 for further validation based on the results of our bioinformatics analysis. As shown in Figures 6B, C, the expression of AK2 was significantly higher in pancreatic cancer tissues compared to para-carcinoma tissues (*p* < 0.05). However, no KEGG terms showed significant enrichment. Conversely, in the low-risk group, the enriched KEGG pathways and GO terms were mainly associated with intracellular signaling and primary immunodeficiency, such as steroid hormone biosynthesis and calcium signaling pathways (Supplementary Figure S2A–B).

### Relationship between risk scores and immune cell infiltration and response to adjuvant chemotherapy

**FIGURE 7:**
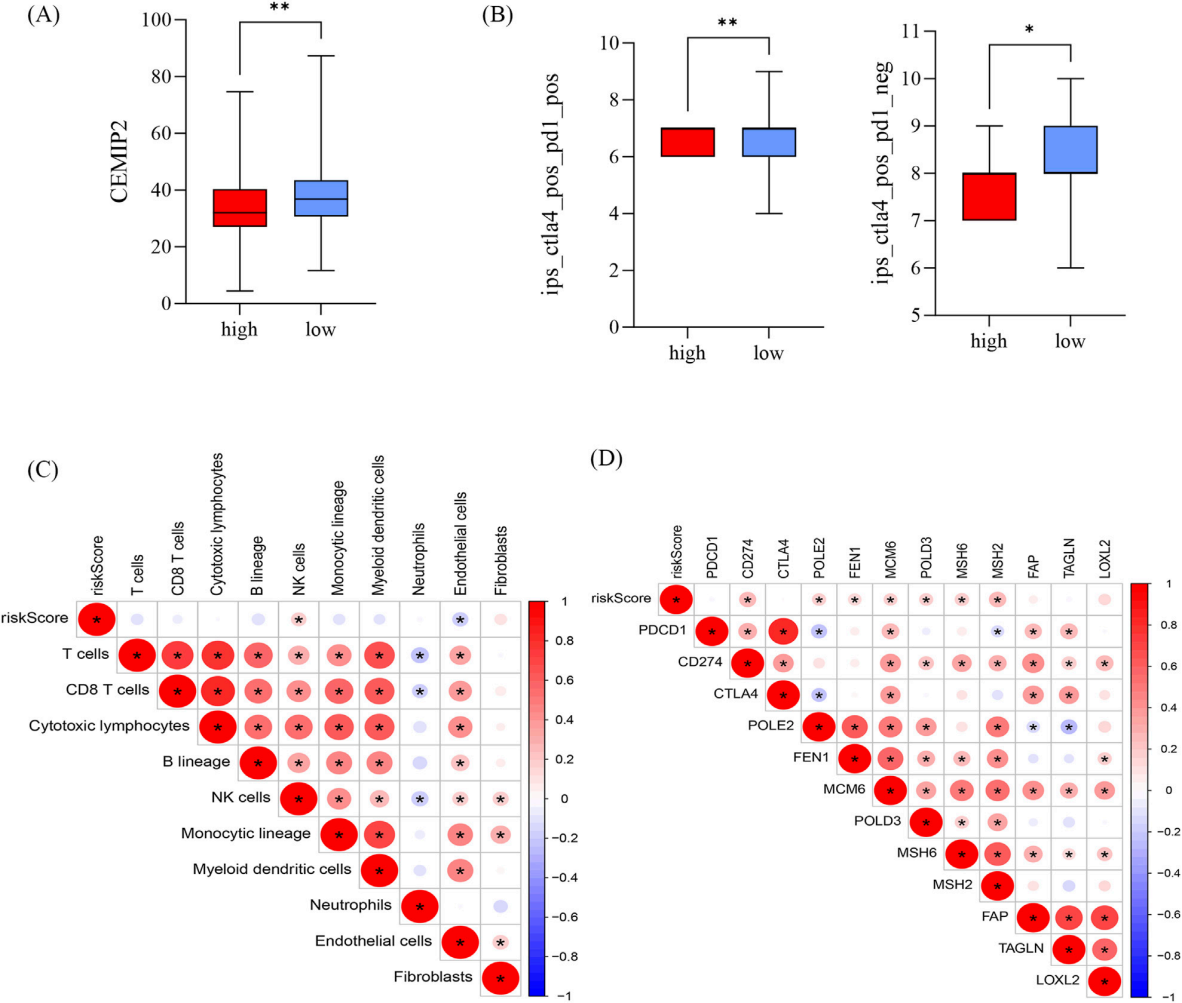
Relationship between risk scores and immune cell infiltration and response to adjuvant chemotherapy **(A)** Difference in CEMIP2 in high and low risk groups. **(B)** IPS scores in high and low risk groups, *p < 0.05, **p < 0.01, ***p < 0.01. **(C, D)** Correlation of risk score with immune cells **(C)** and immune checkpoint-associated genes **(D)**; red represents positive correlation and blue represents negative correlation.

CEMIP2 and NDUFB8 have been shown to be associated with sensitivity to adjuvant chemotherapy in patients with PDAC (Jiang et al., 2024). Further analysis of the relationship between the high-risk and low-risk groups and these two genes revealed that CEMIP2 expression was significantly higher in the low-risk group compared to the high-risk group in advanced PDAC patients (Figure 7A). Conversely, the expression level of NDUFB8 did not show a significant difference between the two groups. The IPS score with CTLA4 blockers, IPS with CTLA4, and PD1 blockers in the low-risk group was significantly higher than in the high-risk group (Figure 7B). Because of the differences in IPS scores between high-risk and low-risk group, we further analyzed the relationship between the risk score and immune cell infiltration to explore the possible reasons for this difference (Figure 7C). In the TCGA cohort, the abundance of neutrophils was significantly higher in the high-risk group, while endothelial cell and T-cell infiltration were significantly reduced (Supplementary Figure S3A). It is well known that the expression levels of immune checkpoint-related genes are closely related to the clinical efficacy and prognosis of immunotherapy in PDAC patients. Therefore, we also investigated the relationship between the risk score and checkpoint-related genes (Figure 7D). CD274, LOXL2, MSH2, POLD3, and POLE2 expression were significantly elevated in the high-risk group (Supplementary Figure S3B). In summary, this risk model can predict the effect of adjuvant therapy in advanced PDAC patients to some extent.

### Comparison with other prognostic models

Three prognostic gene models of pancreatic cancer (Feng et al., 2021; Gu et al., 2021; Luo et al., 2021) were chosen to assess their prognostic ability by comparing the AUC and C-index over different time periods. Table 2 demonstrates that this model had the highest AUC in the ROC curves for 1-, 3-, and 5-year OS. Likewise, when comparing the C-index of the four models, the new model outperformed the other three models (Table 2).

**TABLE 2:** Comparison with other prognostic models.

Models	1-year AUC	3-year AUC	5-year AUC	C-index
IMRG	0.828	0.881	0.947	0.774
Feng	0.723	0.687	0.8	0.672
Luo	0.744	0.757	0.745	0.698
Gu	0.578	0.707	0.803	0.582

## Discussion

PDAC is a highly lethal disease that exhibits significant molecular heterogeneity. Currently, the TNM staging system serves as the primary means for prognostic assessment of PDAC patients. However, this system is insufficient in accurately predicting treatment effectiveness and survival outcomes, particularly among patients in the same stage (van Roessel et al., 2018). The variability within staging can be attributed to tumor heterogeneity. With the advancement of precision medicine in cancer treatment, addressing molecular heterogeneity has become a critical focus in cancer research (Juiz et al., 2019; McGranahan and Swanton, 2017). Thus, there is an urgent need to translate genetic and genomic studies of tumors into prognostic genetic models for clinical use.

In this study, we have developed and validated an 8-gene signature that has a strong prognostic capacity for predicting OS in PDAC. These eight genes are unique and do not overlap with any other prognostic genes in PDAC. To begin, we selected a total of 2,638 genes related to metabolism and immunity from the TCGA and GTEx cohorts. From this group, we identified 1,372 genes that showed significant differential expression at the mRNA level. Subsequently, we performed univariate Cox regression analysis to identify the eight genes that best predicted OS in the training cohort. The AUC values for our model were equal to or greater than 0.7, indicating good prognostic prediction performance not only in the TCGA cohort, but also in the GSE57495 cohort. The high accuracy of this risk model in predicting OS suggests that it can serve as a valuable supplement to the existing TNM staging system for prognostic assessment and treatment decision-making. Additionally, when compared to previous genetic prognostic models for PDAC, the model demonstrated higher AUCs in the 1-, 3-, and 5-year ROC curves. This indicates that this model has better predictive ability and accuracy compared to previous models.

Adenylate kinase (AK) is highly conserved in a wide range of organisms. It is a key enzyme in the monitoring of homeostatic metabolism of cellular adenine nucleotides (Klepinin et al., 2020). Additionally, it plays a crucial part in AK - AMP - AMPK signaling, cell cycle regulation, cell proliferation, and energy transfer during cellular processes, including ATP distribution from mitochondria. Growing evidence suggests that the AK isoform network also controls several cellular processes such as cell motility and cell differentiation (Panayiotou et al., 2014). Adenylate kinase 2 (AK2) is located in the space between the membrane and the slit and is essential for ATP export and mitochondrial nucleotide exchange (Dzeja and Terzic, 2009). A study discovered that the expression of AK2 is upregulated in metastatic pancreatic endocrine tumors, leading to tumor formation (Hansel et al., 2004; Kim et al., 2014). Another study showed that AK2 overexpression activates the TGF-β/Smad3/Smad2/Smad4 signaling pathway, enhancing invasion and affecting the effectiveness of adjuvant therapy through EMT (Cai et al., 2021). Additionally, AK2 has been shown to regulate the survival, proliferation, and effector functions of immune cells by maintaining cellular energetic homeostasis through the conversion of ATP and AMP to adenosine diphosphate. It is crucial for B cell activation and survival and may serve as a potential target for new therapeutic approaches in which B cells play a central role (Campos Codo and Moraes-Vieira, 2020; O'Neill et al., 2016; Six et al., 2015). Based on these findings, AK2 has been selected for further study in this research. Immunohistochemistry of collected pathological section specimens confirmed that the expression level of AK2 is increased in PDAC compared to normal tissues. However, the specific role and mechanism of AK2 in PDAC still require verification through additional experiments.

For patients with PDAC, it is crucial to identify predictive biomarkers that can maximize survival benefits and minimize side effects. This study aimed to determine whether an 8-gene signature score could assist in the development of personalized treatments for PDAC patients. Alongside surgery, adjuvant chemotherapy is a vital treatment option for PDAC patients (Xu et al., 2016). A study conducted by Jiang et al. discovered a strong correlation between DUFB8 and CEMIP2 and the sensitivity to adjuvant chemotherapy. These findings may also apply to neoadjuvant chemotherapy in general (Jiang et al., 2024). In this study, we observed that the expression of CEMIP2 was significantly higher in the low-risk group compared to the high-risk group. According to Jiang’s research, this suggests that the low-risk group exhibited greater sensitivity to adjuvant chemotherapy, particularly to gemcitabine. On the other hand, the expression level of NDUFB8 did not show a significant difference between the two groups. This may be attributed to several factors: the cohorts in this study were relatively small, and there may be differences in patient composition-such as racial composition-between this study and the cohort used in Jiang’s research. Consequently, patients with lower PDAC scores may experience better outcomes with adjuvant chemotherapy than those with higher scores.

In addition to chemotherapy and radiotherapy, immunotherapy and targeted therapies are emerging as important strategies for treating cancer (Colli et al., 2017; Lee et al., 2018; Sung et al., 2021; Sanmamed and Chen, 2019). However, while immunotherapies have been successful in treating other types of cancer, they have not been as effective in pancreatic cancer treatment (Akce et al., 2018; Feng et al., 2017). Research has shown that tumor-infiltrating lymphocytes (TINs) in the tumor microenvironment play a role in promoting tumors and metastasis by suppressing effector T cells and natural killer cells. This suggests that a decrease in the number of TINs may overwhelm anticancer immunity in the PDAC and suggests that it can be used to predict immunotherapy response (Li et al., 2020; Veglia et al., 2021; Zhao et al., 2023) ]. In the TCGA cohort, it was found that the high-risk group had a significantly higher abundance of neutrophils, while the infiltration of endothelial cells and T cells was significantly reduced. Additionally, the high-risk group showed significantly elevated expression of immune checkpoint-related genes CD274, LOXL2, MSH2, POLD3, and POLE2, whereas the low-risk group did not show significant expression of these genes. CD274, also known as PD-L1 (programmed death-ligand 1), is a transmembrane protein encoded by the CD274 gene. It plays a critical role in immune system regulation by binding to its receptor PD-1 (programmed death-1) on T cells. The expression level of CD274 is often used as a potential biomarker to predict the efficacy of patients on PD-1/PD-L1 inhibitors (Gou et al., 2020). Lysyl oxidase-like 2 (LOXL2) is a secreted enzyme involved in extracellular matrix (ECM) remodeling through the cross-linking of collagen and elastin. A Study has shown that LOXL2 promotes fibrosis and enhances immune escape in PDAC, which is an important factor limiting the effectiveness of immunotherapy (Alonso-Nocelo et al., 2023). MSH2, POLD3, and POLE2 are all involved in DNA repair and are often used as markers to predict the efficacy of ICIs. In summary, the prognostic model developed in this study can be useful in guiding personalized immune therapy. For patients with advanced pancreatic cancer, the low-risk subgroup showed significantly higher IPS scores when treated with CTLA4 blockers alone or a combination of CTLA4 and PD1 blockers, compared to the high-risk group. This indicates that the low-risk subgroup may respond better to immunotherapy with these agents. In summary, this model not only has the potential to predict patients’ sensitivity to adjuvant chemotherapy, but also to predict their responsiveness to immunotherapy to some extent. This information can assist clinicians in formulating more optimal individualized treatment strategies.

To further understand why the 8-gene signature is linked to a poor prognosis, we conducted an analysis on functional annotation and pathway enrichment. We found that several oncogenic pathways, which are known to be involved in tumor progression, chemoresistance, and immune cell infiltration, were significantly enriched. These results shed light on how the risk scores can impact patient survival and overall outcomes.

Despite the improved prediction of OS compared to previous models and the ability to predict treatment efficacy to some extent, this study is still limited by its retrospective data and several other factors. First, the clinical utility of genetic prognostic models for PDAC management necessitates validation in additional prospective studies. Second, the relatively small cohort size in this study warrants confirmation of findings in larger, more diverse populations. Finally, further *in vivo* and *in vitro* investigations are essential to elucidate the biological functions and underlying mechanisms of these eight genes, with a particular focus on AK2 in PDAC tumorigenesis. Follow-up studies will be conducted to build upon these findings in future research.

In conclusion, this study introduces an 8-gene prognostic model that enhances predictions of OS and potential response to adjuvant chemotherapy and immunotherapy. We have conducted an initial investigation into the clinical significance and biological relevance of the model, which can contribute to personalized treatment decision-making. However, the predictive accuracy of this prognostic model should be verified in larger cohorts and prospective studies, and further improved through additional relevant *in vitro* or *in vivo* experiments.

## Data Availability

The original contributions presented in the study are included in the article/Supplementary Material, further inquiries can be directed to the corresponding authors.

## Supplementary material

SUPPLEMENTARY FIGURE S1 Heatmap of the significant differential genes.

SUPPLEMENTARY FIGURE S2 GSEA analysis for the the low-risk group.

SUPPLEMENTARY FIGURE S3 Relationship between risk scores and immune cell infiltration **(A)** Endothelial cells, T cells, and neutrophils differed in high and low risk groups. **(B)** MSH2, CD274, LOXL2, and POLE2 differed in high and low risk groups.
