# Supplementary figures and images for: The value of a metabolic and immune-related gene signature and adjuvant therapeutic response in pancreatic cancer
Source: Front Genet. 2025 Jan 3;15:1475378. doi: 10.3389/fgene.2024.1475378 (PMC11758928; doi:10.3389/fgene.2024.1475378)

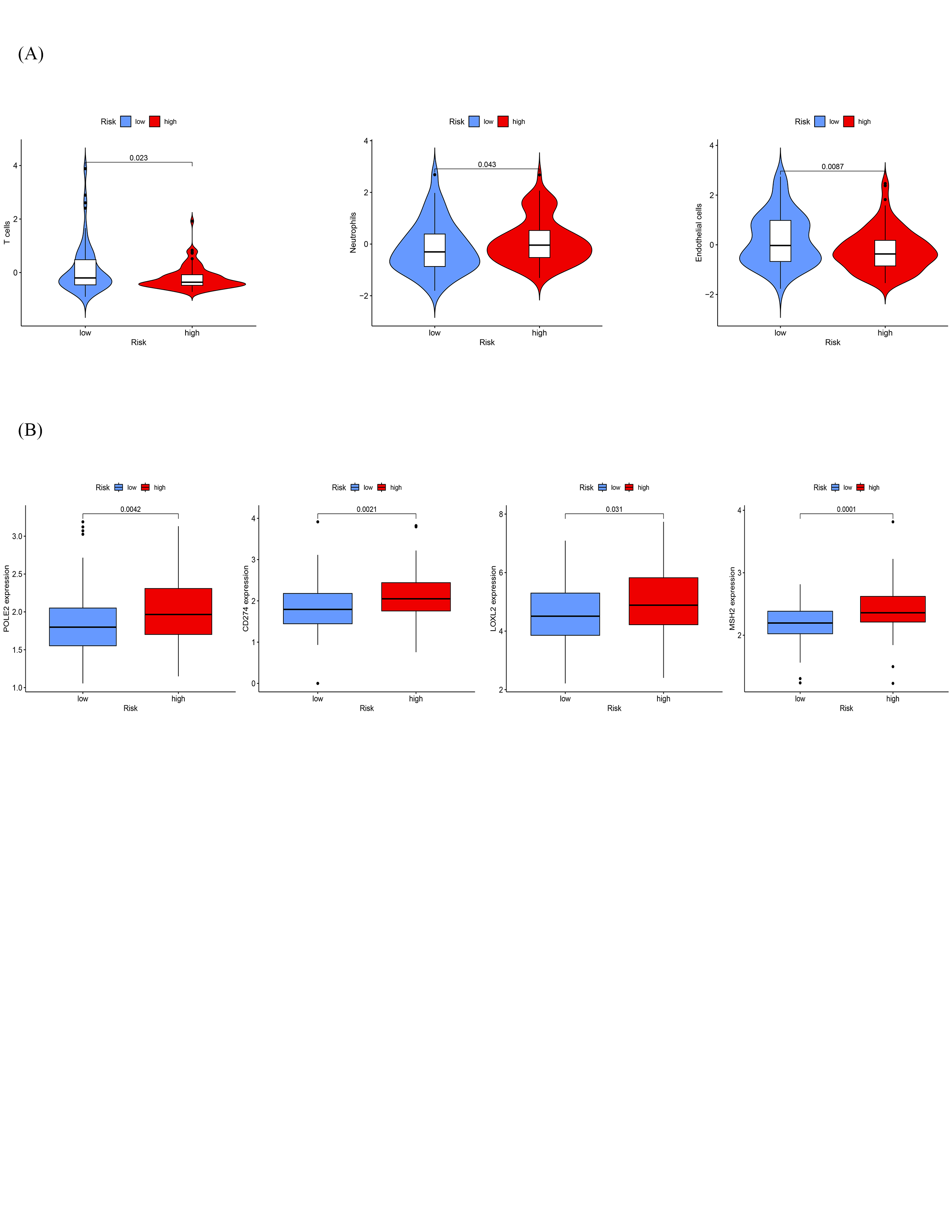

Supplement: Supplementary file 1 [file Image3.tif]

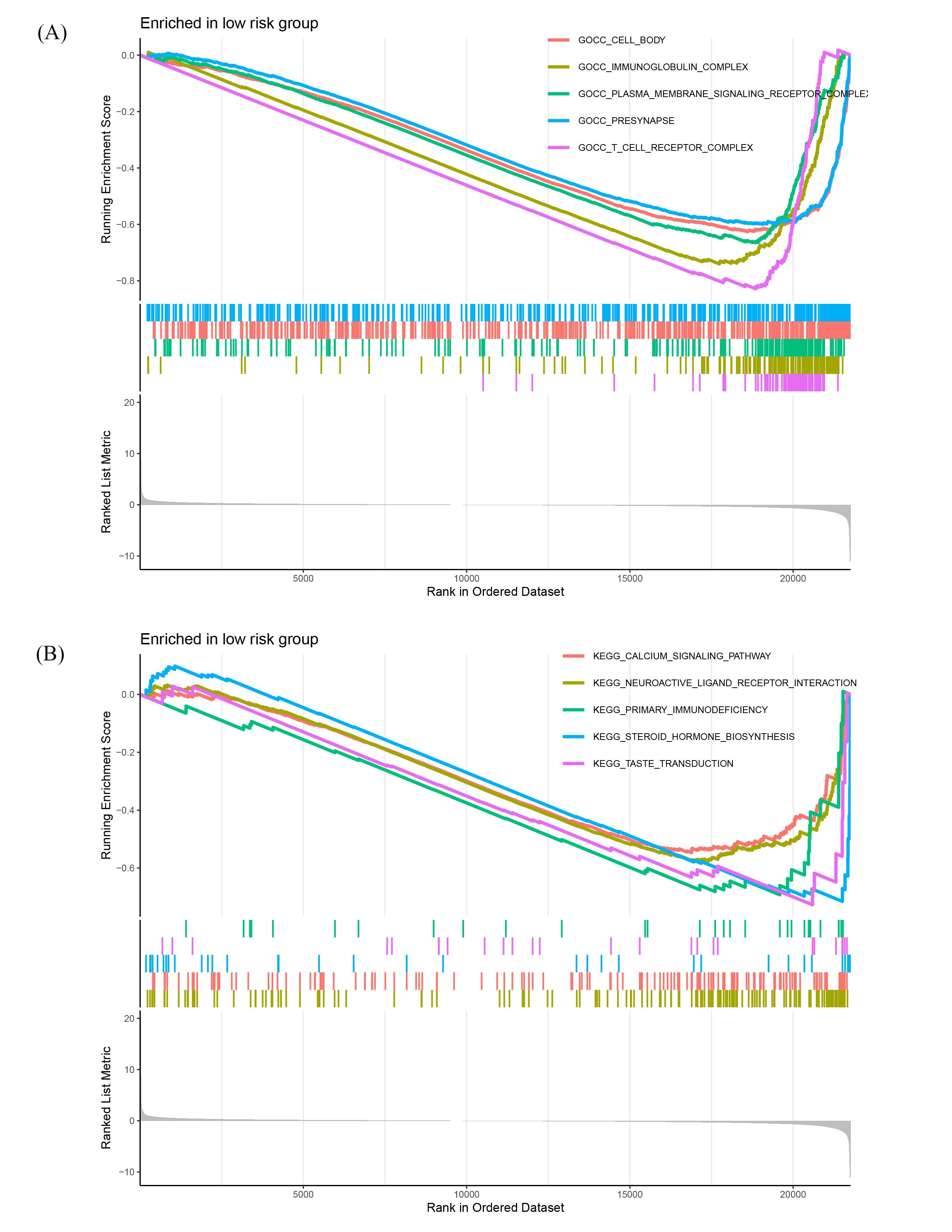

Supplement: Supplementary file 2 [file Image2.tif]

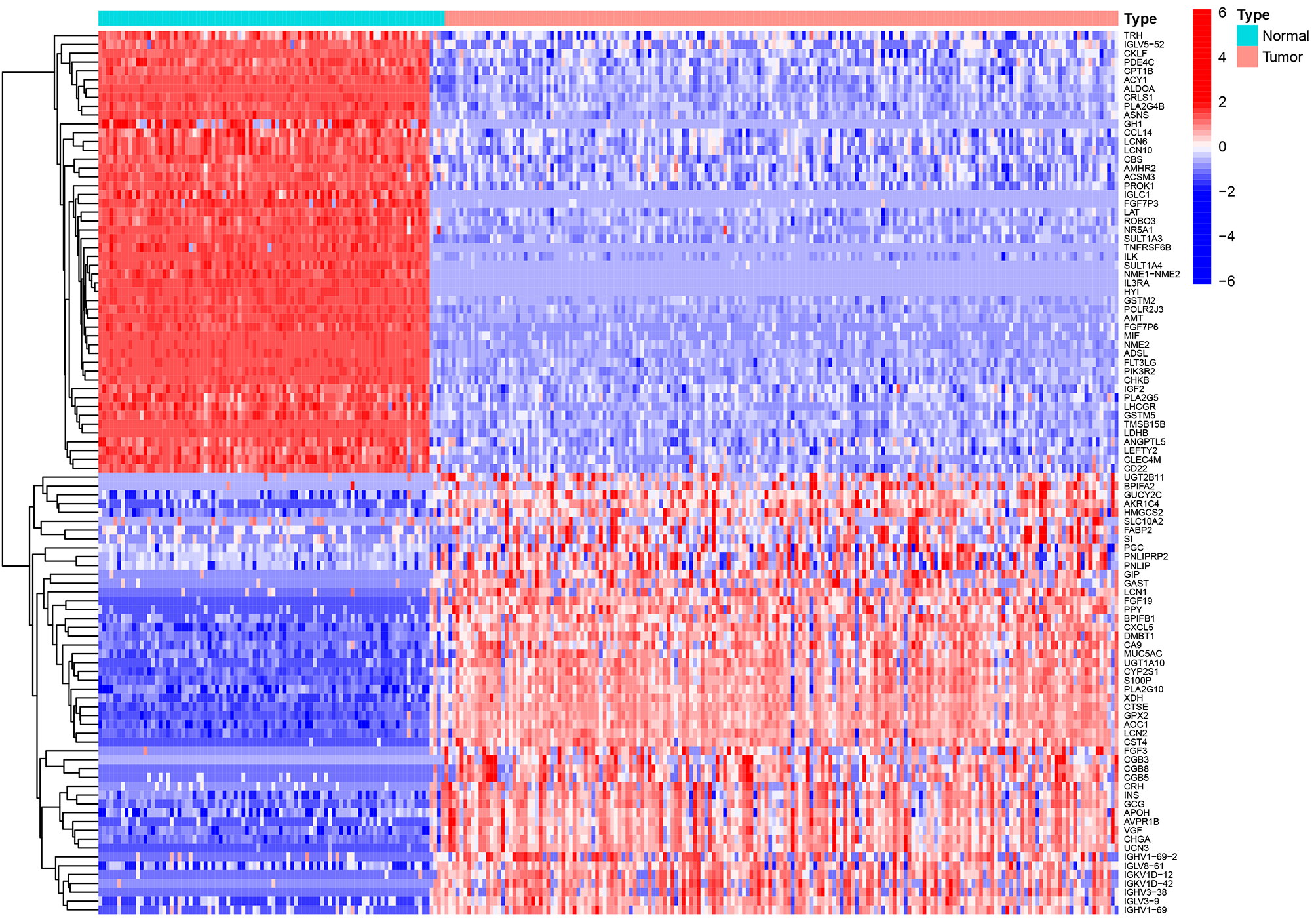

Supplement: Supplementary file 3 [file Image1.tif]
